# Regional Mapping of Brain Glutamate Distributions Using Glutamate-Weighted Chemical Exchange Saturation Transfer Imaging

**DOI:** 10.3390/diagnostics10080571

**Published:** 2020-08-08

**Authors:** Do-Wan Lee, Chul-Woong Woo, Dong-Cheol Woo, Jeong Kon Kim, Kyung Won Kim, Dong-Hoon Lee

**Affiliations:** 1 Department of Radiology, Asan Medical Center, University of Ulsan College of Medicine, Seoul 05505, Korea; dwlee.mri@gmail.com (D.-W.L.); kim.jeongkon@gmail.com (J.K.K.); medimash@gmail.com (K.W.K.); 2 Convergence Medicine Research Center, Asan Institute for Life Sciences, Asan Medical Center, Seoul 05505, Korea; wandj79@hanmail.net (C.-W.W.); dcwoo@amc.seoul.kr (D.-C.W.); 3 Department of Convergence Medicine, Asan Medical Center, University of Ulsan College of Medicine, Seoul 05505, Korea; 4 Department of Radiation Convergence Engineering, Yonsei University, Wonju 26493, Korea

**Keywords:** glutamate, chemical exchange saturation transfer, healthy rat brain, brain regions

## Abstract

Purpose: To investigate glutamate signal distributions in multiple brain regions of a healthy rat brain using glutamate-weighted chemical exchange saturation transfer (GluCEST) imaging. Method: The GluCEST data were obtained using a 7.0 T magnetic resonance imaging (MRI) scanner, and all data were analyzed using conventional magnetization transfer ratio asymmetry in eight brain regions (cortex, hippocampus, corpus callosum, and rest of midbrain in each hemisphere). GluCEST data acquisition was performed again one month later in five randomly selected rats to evaluate the stability of the GluCEST signal. To evaluate glutamate level changes calculated by GluCEST data, we compared the results with the concentration of glutamate acquired from ^1^H magnetic resonance spectroscopy (^1^H MRS) data in the cortex and hippocampus. Results: GluCEST signals showed significant differences (all *p* ≤ 0.001) between the corpus callosum (−1.71 ± 1.04%; white matter) and other brain regions (3.59 ± 0.41%, cortex; 5.47 ± 0.61%, hippocampus; 4.49 ± 1.11%, rest of midbrain; gray matter). The stability test of GluCEST findings for each brain region was not significantly different (all *p* ≥ 0.263). In line with the GluCEST results, glutamate concentrations measured by ^1^H MRS also appeared higher in the hippocampus (7.30 ± 0.16 μmol/g) than the cortex (6.89 ± 0.72 μmol/g). Conclusion: Mapping of GluCEST signals in the healthy rat brain clearly visualize glutamate distributions. These findings may yield a valuable database and insights for comparing glutamate signal changes in pre-clinical brain diseases.

## 1. Introduction

Glutamate is a major excitatory neurotransmitter in the mammalian central nervous system and is involved in neuronal function [1,2,3]. Glutamatergic dysfunction in the brain is implicated in various neuropsychiatric disorders such as epilepsy, Alzheimer’s disease, autism, and aging, as well as neurological issues stemming from gross abnormalities such as ischemia or tumors [4,5,6,7,8,9]. Therefore, detecting in vivo glutamate signal changes in the brain can be applied to the diagnosis and treatment plan of multiple diseases where glutamate changes are involved and may play a role as a biomarker for neurological diseases in vivo.

To date, a variety of studies using medical imaging modalities such as magnetic resonance imaging (MRI), magnetic resonance spectroscopy (MRS), single-photon emission tomography (SPECT), and positron emission tomography (PET) have attempted to image the distribution of in vivo neurotransmitters, particularly glutamate, in the brain [10,11,12,13,14,15]. MR-based imaging and quantification methods have high enough resolution to provide details of brain structure with MRI and to show neurological changes by MRS. Although MRS has sufficient sensitivity to detect neurotransmitters in many areas of the brain, direct quantification of neurotransmitters in certain cases, such as separation of individual pools of glutamate, is limited in clinical MRI systems (≤3 T). However, the use of MRS with an ultra-high field (≥7 T) provides improved sensitivity because of high Larmor frequency and improved chemical shift dispersion, allowing better characterization of individual responses. [16] Moreover, nuclear medicine-based imaging systems such as SPECT and PET use radioactive ligands that bind to and measure glutamate receptors in vivo [14]. Although these glutamate-receptor-specific ligands have high sensitivity in vivo, they are limited in application because of low spatial resolution and widespread availability of facilities for producing labeled isotopes.

Chemical exchange saturation transfer (CEST) MRI utilizes the reduction of the bulk water magnetization through the exchange of saturated magnetization from exchangeable protons of solute metabolites by applying a radio-frequency (RF) pulse and shows enhanced sensitivity compared to other imaging methods [17,18]. It has been used with multiple proton-exchanging solutes such as glutamate, amides, creatine, and glycogen, and applied in multiple brain studies to monitor in vivo changes in solute concentration and exchange-related properties. [19,20,21,22,23] Imaging of in vivo glutamate using glutamate CEST (GluCEST) has been demonstrated in various in vivo brain studies [16,22,24,25,26,27,28,29,30,31,32]. The application of GluCEST not only depicts changes in glutamate associated with brain diseases as described in previous studies [22,24,25,26,27,29,31,32] but also shows the difference between gray matter and white matter in the subcortical area in healthy human subjects [16,28]. Especially since the GluCEST imaging has introduced many pre-clinical studies, including proof-of-principle studies, which have demonstrated differences in brain glutamate levels between disease groups and healthy controls. In various neurological diseases (Alzheimer’s disease, Parkinson’s disease, and Huntington’s disease), the evaluation of glutamate levels has been conducted around specific brain regions (corpus callosum, cortex, or striatum) where each disease is predominantly expressed. Moreover, comparisons between groups (normal vs. disease) have been applied based on those specific regions. Although the neurotypical brains are an essential consideration in which glutamate changes in specific regions by the disease group are observed, it is insufficient to provide a database of normal ranges of glutamate levels for various brain regions at once. Therefore, note that it is necessary to provide a database for evaluating in vivo glutamate levels in various brain regions based on healthy subjects. Moreover, establishing a study for reproducibility of signal measurement in order to implement more extensive pre-clinical GluCEST studies could be valuable.

In this study, we applied GluCEST to healthy rat brains and performed extensive quantitative and qualitative analyses in multiple brain regions to elucidate the in vivo distribution of glutamate. We also performed a stability test between two time points (initial measurement and one month after) to demonstrate the signal stability of GluCEST measurements and also compare ^1^H MRS data from the cerebral cortex and hippocampus to that obtained with the GluCEST signal.

## 2. Materials and Methods

### 2.1. Animal Preparation

All animal experiments were approved by the University Animal Care and Use Committee (approval date: 29-01-19; permit code: 2019-12-023). Eleven Sprague-Dawley rats (male; eight weeks; 250–300 g; Orient Bio Inc., Gyeonggi-do, Korea) were used in this study. Each rat was placed on the imaging bed in the MRI scanner under anesthesia with isoflurane (4% for induction and 2% for maintenance) and in a mixture of 70% nitrous oxide and 30% oxygen supplied via a nose cone. The rat was immobilized using an ear-bar to minimize motion, and a small animal monitor system (SA Instruments Inc., Stony Brook, NY, USA) for respiration with a pressure transducer was attached to the rat’s abdomen. Body temperature was maintained at approximately 37 °C with a warm-water circulating flat-bed.

### 2.2. Data Acquisition

All experiments were performed on a Bruker PharmaScan 7 T scanner (BioSpin GmbH, Bremen, Germany) equipped with a 400 mT/m self-shielding gradient system and an actively decoupled cross-coil setup, which consists of a body coil for RF transmission and 25 mm single-loop surface coil for signal reception. A localization scan was first performed to ensure animal positioning and image quality before acquiring all images. T_2_-weighted images based on a turbo-rapid acquisition with relaxation enhancement (RARE) pulse sequence were collected using the following parameters: time of repetition (TR)/time of echo (TE) = 4000 ms/33 ms; RARE factor = 8; echo spacing = 11 ms; field of view (FOV) = 30 × 30 mm^2^; matrix size = 96 × 96; slice thickness = 1.5 mm; seven contiguous axial slices. One axial slice among the T_2_-weighted images showing multiple brain regions such as cortex, hippocampus, and corpus callosum was used for matching GluCEST imaging with the same FOV, matrix size, slice thickness, and voxel localization of ^1^H MRS. In each rat scan, localized high-order shimming was always applied before GluCEST and ^1^H MRS sequences were initiated. GluCEST imaging was acquired using a turbo-RARE pulse sequence with the following parameters: TR/TE = 4200 ms/36.4 ms, FOV = 30 × 30 mm^2^, matrix size = 96 × 96, slice thickness = 1.5 mm, RARE factor = 16, and echo spacing = 6.1 ms. The GluCEST images were acquired at 25 saturation frequency offsets ranging from −6 to +6 ppm (0.5 ppm increment) using a long, continuous-wave RF saturation pulse (saturation power/saturation time = 3.6 μT/1 s) [22,33], and reference image (S_0_ image) without saturation pulse was acquired. To minimize effects of B_0_ and B_1_ field inhomogeneity, we acquired water saturation shift referencing (WASSR) Z-spectra (33 frequency offsets from −0.8 to +0.8 ppm; 0.05 ppm increment; 0.05 μT RF saturation power) [34], and B_1_ map using the double flip-angle (30° and 60°) method [22]. Additionally, five randomly selected rats among 11 rats were scanned again after one month to evaluate the signal stability of GluCEST imaging.

Based on the data of the stability tests in the five selected rats, ^1^H MRS data acquisition was performed to assess glutamate concentration. Water-suppressed ^1^H MRS single-voxel spectra were acquired from a region of interest (ROI) in the left cerebral cortex (2.0 × 2.0 × 3.0 mm^3^; 12.0 μL) and hippocampus (4.5 × 1.0 × 2.5 mm^3^; 11.25 μL) using a spin-echo-based point-resolved spectroscopy sequence using the variable power and optimized relaxation delays method with the following parameters: TR/TE = 5000 ms/16.3 ms, average number = 128, number of data points = 2048, and spectral width = 5000 Hz.

In each rat, the data acquisition time was 655.2 s, including S_0_ (25.2 s for each frequency offset) for CEST imaging and 22 min 3 s for ^1^H MRS.

### 2.3. Data Analysis

All GluCEST data analysis was processed pixel-by-pixel and performed using an in-house MATLAB 2016a (MathWorks, Natick, MA, USA). GluCEST data were first corrected for B0 field inhomogeneity by the WASSR method [34], which was used to generate a B0 map. Interpolated Z-spectra at a finer resolution of 0.01 ppm with cubic spline in each voxel was used to correct B0 inhomogeneity. The GluCEST map for the imaging slice was generated by relative changes in the percentage units as follows: GluCEST (%) = [(M_sat_ (−Δω) – M_sat_ (+Δω))/M_sat_ (−Δω)] × 100, where M_sat_ (±Δω) is the magnetization acquired with saturation pulse applied at +3.0 and −3.0 ppm to the water resonance, respectively [22,33]. Subsequently, GluCEST contrast was corrected at each voxel using relative B_1_ values, as described in the previous study [22]. For the quantitative analysis of GluCEST, eight ROIs were carefully drawn in the cerebral cortex, hippocampus, corpus callosum, and the rest of the midbrain regions of the left and right hemispheres.

Spectral fitting and quantification of ^1^H MRS data were performed with a fully blind spectral process using the Linear Combination Model software (LCModel, v. 6.2–1L, copyright: Stephen W. Provencher) with a simulated basis set including 18 metabolites as follows: alanine (Ala), aspartate (Asp), myoinositol (mIns), creatine (Cr), gamma-aminobutyric acid (GABA), phosphocreatine (PCr), glutamate (Glu), glutamine (Gln), glucose (Glc), scyllo-inositol (sI), glycine (Glyc), glycerophosphocholine (GPC), lactate (Lac), N-acetylaspartylglutamate (NAAG), N-acetylaspartate (NAA), glutathione (GSH), phosphocholine (PCh), taurine (Tau), total NAA (tNAA) = NAA + NAAG, Glx = Glu + Gln, total Cr (tCr) = Cr + PCr, and total Cho (tCho) = GPC + PCh. All signal intensities from the in vivo basis set were processed with water scaling and eddy current correction, and metabolite concentrations were obtained (μmol/g). All metabolite peaks were fitted in the chemical shift range from 4.0 to 0.3 ppm. The ^1^H MRS data analysis was performed in the left cerebral cortex and hippocampus of each rat.

### 2.4. Statistical Analysis

Statistical differences in signal intensities of GluCEST between the left and right hemisphere of each brain region and those for stability testing were analyzed by the Wilcoxon signed-rank sum test with a *p*-value below 0.05. For averaged GluCEST values in both hemispheres at four ROIs, a non-parametric Kruskal–Wallis H test followed by post-hoc analysis for pairwise comparison was performed with a Wilcoxon rank-sum test to account for multiple comparisons. In this statistical analysis, *p* < 0.0083 (=0.05/6) was considered significant. All statistical analysis was performed using PASW statistics software (version 18.0; SPSS Inc., Chicago, IL, USA).

## 3. Results

**Figure 1:**
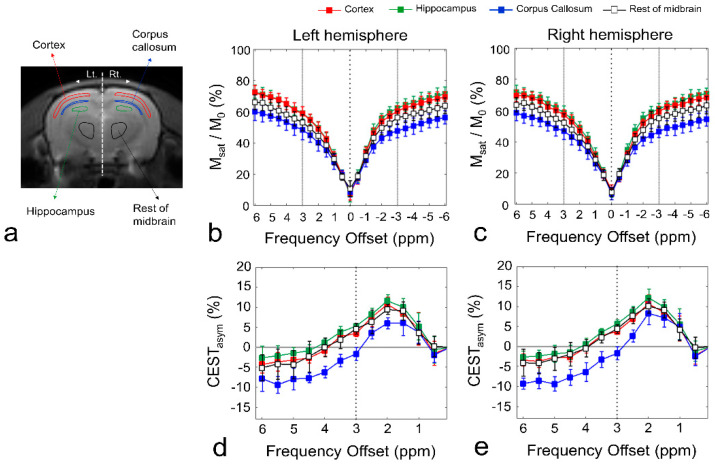
Regions of interest (ROIs) for quantitative analysis of chemical exchange saturation transfer (CEST) data (**a**). Z-spectra (**b**,**c**), and asymmetry curves (**d**,**e**) for each ROI in the left hemisphere and right hemisphere. All square data points indicate mean values (*n* = 11), and error bars represent standard deviation. The Z-spectra and calculated CEST asymmetry curves in each ROI are shown in different colors (red, cortex; green, hippocampus; blue, corpus callosum; black, rest of midbrain). The black dotted line in the asymmetry curve (**d**,**e**) represents glutamate CEST (GluCEST) signal at 3.0 ppm.

Figure 1 shows the defined ROIs of the brain regions (Figure 1a), Z-spectra (Figure 1b,c), and CEST asymmetry (CEST_asym_) curves (Figure 1d,e) in ROIs for left and right hemispheres. In both hemispheres, the Z-spectra, giving the ratios of water signal intensities with (M_sat_) and without (M_0_) saturation, showed overall higher signal intensities for all frequency offsets in the gray matter regions than those in the white matter regions. The signal attenuation difference was mainly due to the direct water saturation (DS) close to the water frequency and a semi-solid MT effect over the whole spectral range. In addition, the visible upward shifts in the Z-spectra in the gray matter regions are due to the narrowing of the DS curve caused by the difference in relaxation time (T_2_). All CEST_asym_ curves (*n* = 11) in gray matter regions (cortex, hippocampus, and rest of midbrain) and white matter (corpus callosum) showed a maximum asymmetry at ~2 ppm, and the curves in gray matter regions had higher asymmetry values than those in the white matter region at both hemispheres. In addition, as indicated in previous studies, the broadness phenomenon in asymmetry curves can be partially attributed to potential amine CEST effects such as creatine and chemical shift average effects due to the fast exchange rate of glutamate, which shifts the line peak toward water resonance [16,35].

**Figure 2:**
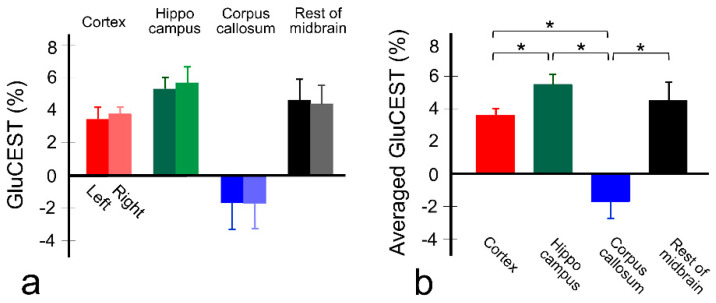
Quantitative values of GluCEST signal in each brain region (*n* = 11) in both hemispheres (**a**) and averaged values between both hemispheres (**b**). The bars represent mean values and error bars represent standard deviations. * *p* < 0.0083 (=0.05/6) via a non-parametric Kruskal–Wallis H test with pairwise comparison using Wilcoxon rank-sum test.

Figure 2 shows the GluCEST signals (*n* = 11) in the ROIs of each hemisphere (Figure 2a), and averaged GluCEST signals between both hemispheres in the gray and white matter regions (Figure 2b). Overall, the GluCEST signals in all gray matter regions are higher than in the white matter region, as shown in Figure 1, and all GluCEST signals calculated from the left and right hemispheres were not significant as follows: left and right hemisphere, respectively; 3.43 ± 0.69% and 3.76 ± 0.40% in cortex (*p* = 0.286), 5.28 ± 0.70% and 5.66 ± 0.98% in hippocampus (*p* = 0.374), −1.69 ± 1.13% and −1.72 ± 1.08% in corpus callosum (*p* = 0.790), and 4.59 ± 1.29% and 4.38 ± 1.12% in hippocampus (*p* = 0.594). In the averaged GluCEST signals, the signal calculated in the hippocampus (5.47 ± 0.61%) is highest among the gray matter regions, followed by the signals in the rest of the midbrain (4.49 ± 1.11%) and cortex (3.59 ± 0.41%). Statistical significance was achieved between cortex and hippocampus (*p* ≤ 0.0002) in gray matter regions, but not between the rest of the midbrain and hippocampus (*p* = 0.013), and cortex (*p* = 0.019). The GluCEST signal in the white matter region (−1.71 ± 1.04%) showed statistical significance over all gray matter regions (all *p* ≤ 0.0001).

**Figure 3:**
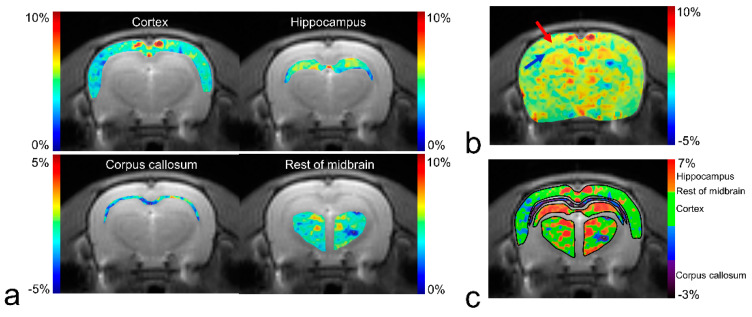
GluCEST maps for manually segmented brain regions (**a**) and whole-brain (**b**) using default color-scale bar (“Jet”) in MATLAB, and GluCEST map for segmented brain regions using a modified discrete color-scale bar (**c**) from a representative rat. The red arrow indicates the cortex, which is a representative gray matter region, and the blue arrow indicates the corpus callosum, which is a white matter region.

Figure 3 shows a GluCEST map of manually segmented brain regions (Figure 3a) and the overall brain area (Figure 3b,c) overlaid on the anatomical image in a representative rat. Although there is a clear contrast between the gray matter and white matter (red and blue arrows in Figure 3b) in overall brain regions, the contrast between gray matter regions is relatively small, as shown in Figure 2 (3.5–5.5% from cortex to hippocampus). Reconstructed GluCEST maps with all segmented brain regions using a modified discrete color-scale bar (based on the average signal for each region) show a distinct contrast in all brain regions (Figure 3c).

**Figure 4:**
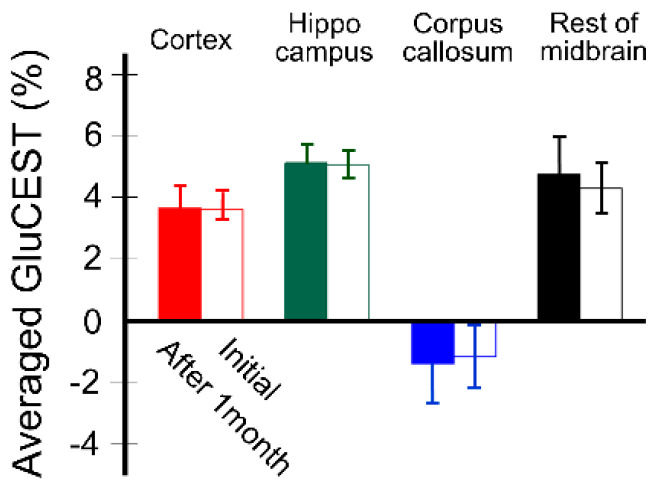
Quantitative values of GluCEST between two time points (from initial scan to one month after) using the same rats (*n* = 5) for signal stability evaluation. The bars in each time point represent averaged values between both hemispheres, and the error bars represent standard deviations.

Figure 4 shows the stability results of quantitative GluCEST signals (*n* = 5) for each brain region. GluCEST signals measured after one month in all brain regions using the same rats were not significantly different from those initially measured, as follows: after one month and initial, respectively; 3.67 ± 0.71% and 3.61 ± 0.34% in cortex (*p* = 0.889), 5.12 ± 0.56% and 5.06 ± 0.47% in hippocampus (*p* = 0.674), −1.39 ± 1.32% and −1.15 ± 1.01% in corpus callosum (*p* = 0.674), and 4.76 ± 1.19% and 4.30 ± 0.80% in hippocampus (*p* = 0.263).

**Figure 5:**
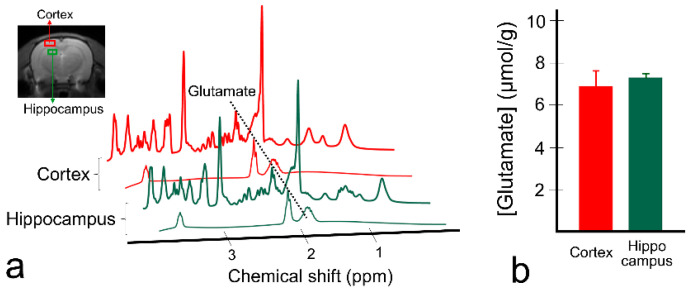
Fitted spectra results of proton magnetic resonance spectroscopy (^1^H MRS) in two brain regions (cortex and hippocampus) from a representative rat (**a**) and quantified glutamate concentrations from the rats (*n* = 5) scanned with ^1^H MRS (**b**). A black-dotted line was placed on the glutamate peak at 2.35 ppm. The bars represent mean values of glutamate concentration, and error bars represent standard deviations.

Figure 5 shows the spectra results of ^1^H MRS in the cortex and hippocampus (Figure 5a) in a representative rat and calculated glutamate concentrations (*n* = 5; Figure 5b). In the spectra fitted using LCModel, glutamate is separated from the other brain metabolites, and the highest peak at 2.35 ppm is clearly visible in both regions. Glutamate concentration was 7.30 ± 0.16 μmol/g in the hippocampus and 6.89 ± 0.72 μmol/g in the cortex, which is in line with the GluCEST results in Figure 2.

## 4. Discussion

GluCEST is a powerful molecular MR imaging technique that reveals changes in glutamate levels in vivo without specialized equipment and has been applied in the diagnosis of many brain diseases such as tumors, ischemia, and psychiatric disorders. GluCEST imaging can be readily and stably performed while evaluating imaging stability and studies about in vivo glutamate distribution in healthy human subjects, as well as providing a database of glutamate changes in disease [16,28,35]. Previous studies using GluCEST imaging in healthy human brains demonstrated a higher contrast in gray matter than in white matter [16,28]. In addition, Kogan et al. showed glutamate distributions in the human spinal cord, which also demonstrated higher GluCEST signals in gray matter compared to white matter [35]. They indicated that higher glutamate distribution in gray matter correlates with higher glutamate concentration due to neuronal density differences between gray and white matter [35,36]. These GluCEST imaging results have also been well-documented through imaging and quantification in studies using PET imaging systems and ^1^H MRS [16,22,37,38].

In this study, we demonstrated the distribution of glutamate in the healthy rat brain by considering the importance of GluCEST imaging and the comparative studies with disease groups in many pre-clinical studies to produce a preliminary database for pre-clinical studies. The aim of this study was three-fold: (i) to investigate the ability for discrimination of GluCEST signals among brain regions, especially between gray and white matter regions, and its differences in the same regions in both cerebral hemispheres; (ii) to evaluate GluCEST signal stability; (iii) to investigate differences in glutamate concentration in brain regions using ^1^H MRS. Our results showed that the GluCEST signal was significantly different between the gray and white matter regions and showed subtle differences between GluCEST signal and glutamate concentrations evaluated by ^1^H MRS between the gray matter regions, as indicated in previous studies [16]. Contrast differences are also indicated through brain mapping results. Our results for the stability of GluCEST imaging showed no significant change in mean values between the initial scan and one month later (all *p* > 0.26), indicating that changes in the GluCEST signal are rare. The calculated coefficients of variation were lower in the cortex (0.15) and hippocampus (0.10) than in the other brain regions (0.89 at corpus callosum and 0.22 at rest of mid-brain). The relatively large coefficients of variation in these regions, as can be observed in large standard deviation ranges, are likely due to the following reasons: (i) partial volume effects on the ROI due to the relatively small and thin anatomical structure of the corpus callosum region observed in our experiment, and (ii) the ROI signal change is relatively large due to the influence of mixed anatomical structures in the rest of the midbrain region. Nonetheless, repeated GluCEST measurements in the same brain regions yield similar signal levels, demonstrating reproducible measurements in terms of quantification and stability of pre-clinical in vivo brain GluCEST imaging.

Since the signal formation and analysis process of GluCEST imaging may be affected by saturation pulse, signal normalization, and other metabolic and biologic effects, it is necessary to mention these effects to our experiments in this study. We performed all experiments on normal rats in the physiological range, at pH 7.0–7.2 [39] with high B_1_ power (3.6 μT) and short duration (1 s) of RF saturation pulse setting in a 7 T MRI system. Although higher GluCEST sensitivity may be achieved with increasing B1 power, the RF saturation parameters used in this study were selected considering the higher glutamate exchange rate and RF specific absorption rate (SAR) mentioned in the previous studies using a 7 T MRI system [22]. Nevertheless, as our results showed, negative CEST_asym_ is observed, presumably due to the underlying MT effects and immobile lipid signals. Therefore, these factors are combined in the CEST_asym_ curves, resulting in a reduction of specificity and sensitivity of quantified GluCEST signals. In addition to the negative CEST_asym_ signals, small variations in glutamate concentrations in the corpus callosum and cortex, as observed in previous biological studies [40,41], and different results of glutamate concentration changes between the hippocampus and cortex via ^1^H MRS and GluCEST can indirectly indicate that the GluCEST signal may be affected by the aforementioned factors, leading to misleading results. The assessment of totally pure glutamate concentration changes by GluCEST imaging is difficult because of the complexity of the various factors. However, note that there is a clear contrast between gray and white matter regions, considering that many published studies [22,26,28,29,33] still demonstrate that glutamate contributions to GluCEST are relatively dominant to other factors. Moreover, by performing normalization of the GluCEST signal using a signal at −3.0 ppm, the dynamic range of the CEST signal may be increased [22,33]. There was no evidence of change in GluCEST signal caused by a pH change due to acidification or basification in the brain.

The present study was performed at single field strengths of 7 T and applied in the 2D single-slice GluCEST imaging. As previous studies indicated, slow to intermediate exchange rate condition is not fulfilled in lower-field strength MRI systems due to the fast exchange rate of glutamate, resulting in low sensitivity of the GluCEST signal [22]. Therefore, applications of GluCEST imaging at 7 T in clinical and pre-clinical settings are recommended and provide adequate signal sensitivity. However, there is a limitation to increasing the field strength in clinical systems because of the SAR issues, so further investigations of glutamate distribution in brains of small animals using a pre-clinical system with a higher field strength (≥9.4 T) may extend our results to establish a wider database for the regional distributions of glutamate in a pre-clinical setting. Moreover, two previous studies reported that gray matter has a higher GluCEST signal (~10%) than does white matter (~8%) in the healthy human brain. These results are consistent with our result in terms of showing clear contrast differences between gray and white matter; however, the signal levels are different. Although a similar saturation power and time were applied, it is meaningful to understand the cause of the difference. As we set multiple ROIs in a single 2D slice, which includes various brain regions, drawn ROIs are limited to small anatomical structures in the brain (such as the corpus callosum in this study) because of partial volume effects. As we tried to draw ROIs in the regions without any involvement, they still have the potential to be affected by pixel sizes. In addition, it may be caused by differences in the receiver coils and their sensitivity since we have used a single-loop surface coil in this study. Further data acquisition using a multi-channel array coil is expected to overcome the limitation of the current coil setup and improve the spatial resolution of images as well as the sensitivity. Moreover, in further clinical translation research, the application of the parallel imaging through the multi-channel phased array coil or the combination with the compressed sensing technique can shorten the scan time [42,43]. These techniques have already been used in the data acquisition of the CEST MRI, and they can be easily applied to the GluCEST imaging in the clinic. Besides, the calculated signal may be misleading because of differences in the molecular biological environment in vivo, which may arise between humans and animals, resulting in slightly different semi-solid MT and other factors affecting GluCEST signals. Nevertheless, as we mentioned earlier, our results demonstrate a clear signal contrast between gray and white matter. Furthermore, a comparison with a previous study on GluCEST imaging in the rat brain at 7 T showed that the quantitative signal levels in the hippocampus (~5.2%) and cortex (~3.5%) are comparable to the results of this study [31]. It would be prudent in future studies to increase scan points with multiple cross-sectional slices or 3D data acquisition for GluCEST imaging analysis to evaluate signals in the extended brain regions. In terms of the use of high B_1_ power in this study, while it may reduce the asymmetric MT effect [33], it cannot completely ignore and reduce other effects on CEST signals such as direct water saturation, semi-solid MT effects, and amines in proteins as previous studies have indicated [44,45]. To minimize these effects on the measured GluCEST signal, research has been applied with multiple fitting methods of the spectra [44,45]. In the future, more exchange-specific quantification of the GluCEST signal through the application of these approaches will be required to produce more valuable results. Furthermore, in the previous GluCEST studies [27,29], researchers mentioned how GluCEST signal changes per mM change through the concentration of glutamate obtained through ^1^H MRS. These studies showed that each mM changes in glutamate concentration, corresponding to 1.3 ~ 1.7% change in the GluCEST contrast. In the current study, the difference in glutamate concentration calculated by ^1^H MRS is ~0.4 μmol/g while the difference in GluCEST is ~1.88%, which is higher compared with previous results [27,29]. This phenomenon may be due to the relatively small sample size of the ^1^H MRS (*n* = 5). In terms of providing the appropriate results of ^1^H MRS, it is difficult to show a statistical significance due to the small sample size, although the results of ^1^H MRS may reflect a similar tendency to GluCEST results in the cortex and hippocampus. In addition, the partial volume effect and the use of different saturation parameters of GluCEST are considered to affect the results, as previous studies indicated [35,44]. Nevertheless, considering GluCEST signals were noticeably different among the brain regions as well as the regions between white matter and gray matter, corresponding regions of ^1^H MRS also showed a similar trend despite the limited sample size. However, future studies are required for a more accurate quantification of ^1^H MRS and GluCEST to examine beyond the erroneous factors.

## 5. Conclusions

We demonstrated in vivo glutamate distribution in the healthy rat brain, with clear signal differences and mapping results between gray and white matter regions. Investigation of GluCEST signals in the healthy rat brain may provide important data to those researching brain diseases, which may show clear changes in glutamate distribution, in terms of providing a primary pre-clinical database for in vivo glutamate distribution. Our results can also be a useful background resource for the further transition to clinical research with GluCEST imaging.
